# PULSE-I - Is rePetitive Upper Limb SEnsory stimulation early after stroke feasible and acceptable? A stratified single-blinded randomised controlled feasibility study

**DOI:** 10.1186/s13063-019-3428-y

**Published:** 2019-07-01

**Authors:** Kausik Chatterjee, Rachel C. Stockley, Steven Lane, Caroline Watkins, Katy Cottrell, Brenda Ankers, Sioned Davies, Mary Fisher Morris, Nick Fallon, Turo Nurmikko

**Affiliations:** 1 Countess of Chester Hospital Foundation Trust, Liverpool Rd, Chester, CH2 1UL UK; 2 Stroke Research Team, School of Nursing, University of Central Lancashire, Preston, PR1 2HE UK; 3 Department of Biostatistics, University of Liverpool, Liverpool, L69 3GL UK; 4 MemCheck Memory Clinic, Beehive Healthcare, Northgate Avenue, Chester, CH2 2DX UK; 5 Department of Psychological Sciences, University of Liverpool, Liverpool, L697ZA UK; 6 Neuroscience Research Centre, The Walton Centre NHS Foundation Trust, Liverpool, L9 7LJ UK

**Keywords:** Stroke, Upper limb rehabilitation, Repetitive sensory stimulation

## Abstract

**Background:**

Reduction in sensorimotor function of the upper limb is a common and persistent impairment after stroke, and less than half of stroke survivors recover even basic function of the upper limb after a year. Previous work in stroke has shown that repetitive sensory stimulation (RSS) of the upper limb may benefit motor function. As yet, there have been no investigations of RSS in the early-acute period despite this being the time window during which the neuroplastic processes underpinning sensorimotor recovery are likely to occur.

**Methods:**

A single-blinded, stratified, randomised controlled feasibility study was undertaken at two NHS acute trusts to determine the recruitment rate, intervention adherence, and safety and acceptability of an RSS intervention in the early period after stroke. Participants were recruited within 2 weeks of index stroke. Stratified on arm function, they were randomised to receive either 45 min of daily RSS and usual care or usual care alone (UC) for 2 weeks. Changes from baseline on the primary outcome of the Action Research Arm Test (ARAT) to measurements taken by a blinded assessor were examined after completion of the intervention (2 weeks) and at 3 months from randomisation.

**Results:**

Forty patients were recruited and randomised (RSS *n* = 23; UC *n* = 17) with a recruitment rate of 9.5% (40/417) of patients admitted with a stroke of which 52 (12.5%) were potentially eligible, with 10 declining to participate for various reasons. Participants found the RSS intervention acceptable and adherence was good. The intervention was safe and there were no serious adverse events.

**Conclusions:**

This study indicates that recruitment to a trial of RSS in the acute period after stroke is feasible. The intervention was well tolerated and appeared to provide additional benefit to usual care. In addition to a definitive trial of efficacy, further work is warranted to examine the effects of varying doses of RSS upon arm function and the mechanism by which RSS induces sensorimotor recovery in the acute period after stroke.

**Trial registration:**

ISRCTN, registry no: ISRCTN17422343; IRAS Project ID: 215137. Registered on October 2016

**Electronic supplementary material:**

The online version of this article (10.1186/s13063-019-3428-y) contains supplementary material, which is available to authorized users.

## Background

Over 15 million people experience a stroke each year worldwide and, in high-income countries, stroke is the single main cause of acquired disability [1]. There are more than 1.2 million stroke survivors living in the UK with over 100,000 new cases of stroke each year [2].

Advances in acute care have dramatically reduced stroke mortality [3] but recovery of sensorimotor function of the upper limb remains problematic. Whilst two thirds of stroke survivors go on to walk independently, less than 20% recover full upper limb function and over half do not regain basic functions of the upper limb after several years [4, 5].

Completing even simple Activities of Daily Living (ADLs) often requires a substantial level of upper limb ability and so persistent impairments in upper limb function produce negative effects upon daily functioning and significantly reduce independence [6, 7]. Consequently, improving upper limb function is a core element of stroke rehabilitation [8]. Current treatment guidelines emphasise that rehabilitation should include high numbers of repetitions of motor tasks (repetitive task training, RTT) to improve sensorimotor function after stroke [9]. Recent work has also identified a 5-week critical window after stroke in which most of the neuroplasticity that underpins recovery of sensorimotor control of the upper limb occurs [10]. This period presents a short but sensitive phase of increased responsiveness to rehabilitation after stroke. It also indicates that the intensity of training is likely to be key in this 5-week period to maximise neuroplastic processes and optimise the recovery of the upper limb. However, in practice, delivering high-intensity RTT in the acute and early subacute period after stroke is challenging. Difficulties arise as it requires participants to be consistently and highly motivated, and rehabilitation staff need to have the time and resources to support RTT [11, 12].

Consequently, there is a clear and urgent need to develop and evaluate new treatments. Such treatments need to be delivered in the early, sensitive period after stroke, must not require significant increases in staff time, cannot be reliant on consistently high levels of motivation in people after stroke, and be able to be used by people with severe hemiparesis.

Repetitive sensory stimulation (RSS) is a largely passive treatment which has been recognised in healthy people to produce neuroplastic changes, similar to those elicited by repetitive task training. [13]. These include lasting changes in corticospinal excitability which may be elicited via a GABA-ergic disinhibition and long-term potentiation produced by glutaminergic mechanisms [14].

RSS interventions have predominantly been evaluated in studies of people many months or even years after stroke [15–19] with benefits to sensation, arm and hand function. Recently, a small randomised, sham-controlled trial evaluating a 2-week RSS intervention in people commenced in the early subacute stage [20] (at least 3 or 4 weeks) after stroke showed significant benefits to sensorimotor function including tactile discrimination and global hand function [21]. However, no studies have used RSS in the acute/very early subacute period (first few days or weeks) after stroke, despite this being likely to be the optimal period for recovery of sensorimotor function [10]. However, there may be practical factors which influence the feasibility and acceptability of using the RSS in the first few days after stroke and of recruiting to, and conducting, a trial of its effectiveness during this period. Therefore, a study was conducted to determine: the feasibility and acceptability of using RSS in the first 2 weeks after stroke (acute and early subacute period) [20]. Collectively this information will inform a future, adequately sized, randomised controlled clinical trial of RSS early after stroke.

## Methods

### Study design

The study was designed as a single-blind, stratified, randomised controlled trial, designed and funded to recruit and follow-up 40 patients within 1.5 years. Patients were recruited at stroke units at the Countess of Chester Hospital NHS Foundation Trust between January and November 2017 and at Basildon and Thurrock University Hospitals NHS Foundation Trust from September to November 2017. Ethical approval was obtained from North West-Liverpool Central Research Ethics Committee (ref no: 16/NW/07/71). The recruitment was stopped as it enrolled the required number of patients.

### Participants

Participants were included if they were aged over 18 years and had suffered a unilateral, confirmed stroke in the past 2 days to 2 weeks, which had left them with sensorimotor deficits of their arm. Those who did not have a National Institute of Health Stroke Scale (NIHSS) arm motor score of between 1 and 4 (NIHSS arm score ranges from 0: no weakness to 4: no movement) and/or a pre-stroke modified Rankin Scale score (mRS) of between 0 and 3 (where 0 = no disability, 3 = moderate disability, requiring some help, but able to walk unassisted) were not included [22–25]. Both of these tools were chosen as they are widely recognised in both clinical and research settings and are recommended by the Stroke Rehabilitation Research Roundtable [26]. Potential participants were also excluded if they had epilepsy, a permanent pacemaker, dermatitis or oedema of the affected hand or if they could not give verbal or written consent.

After going through the inclusion and exclusion criteria, all eligible patients were invited by a Good-Clinical-Practice-trained healthcare professional to take part in this trial. As this was a feasibility trial, only those who could provide a signed informed consent or witnessed verbal consent were allowed to participate in the trial.

#### Stratification and randomisation

After giving informed consent, participants were randomised to either the experimental group comprising 45 min of RSS delivered daily for 2 weeks via a glove plus usual care (RSS) or usual care (UC) alone. Randomisation was stratified by both NHS trust and the patient’s NIHSS arm score (1–2; 3–4). Block randomisation with block sizes of 2, 4 and 6 were used to generate the randomisation lists for each trust and NIHSS arm score. Group allocations were placed in serially numbered sealed envelopes to be opened after consenting; this was done by the trial statistician. Each trust had two randomisation lists, one for each NIHSS are score strata. Researchers undertaking recruitment and randomisation had no prior knowledge or involvement in the generation of the randomisation lists.

#### Interventions

**Fig. 1:**
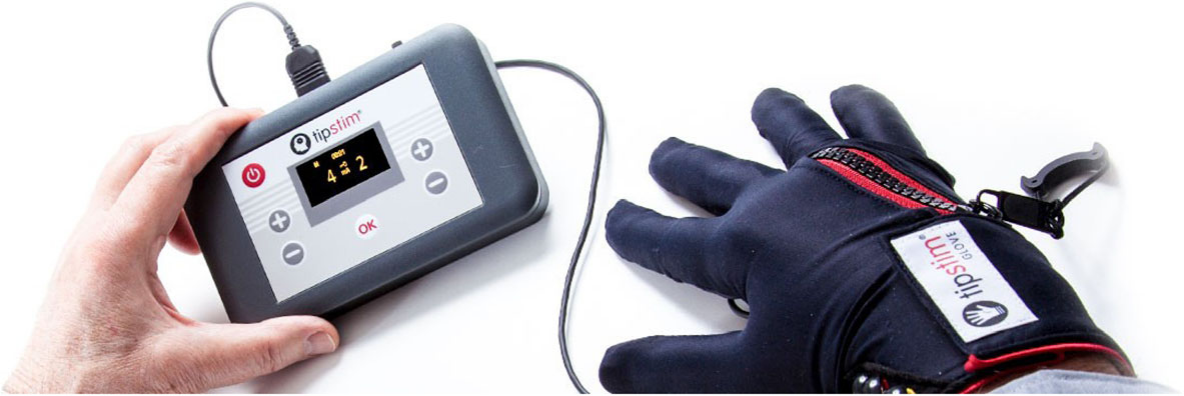
Picture of the repetitive sensory stimulation (RSS) glove and simulator in situ

The RSS group were provided with an appropriate-sized glove and stimulator box (Fig. 1). The RSS glove was placed on the affected hand by the participant with aid from a rehabilitation assistant and/or a family member, as required. Supra-sensory pulses were delivered at a frequency of 20 Hz with an intensity of 1 to 20 mA by electrodes within each glove positioned on the distal and proximal phalanges, providing stimulation to all fingers. The intensity of the current was increased to the highest level that the participant could tolerate and, once this intensity was reached, the participant received 45 min of stimulation. This duration was chosen as 30 min of supra-sensory hand stimulation has been shown to increase cortical excitability, which plateaus by 45 min [27–29]. RSS was repeated daily for 2 weeks (14 sessions, total time: 630 min) [21].

Usual care (UC) comprised a range of individually tailored interventions (necessary for the individual patient) delivered by physiotherapists and occupational therapists who were specialised in neurological rehabilitation. The RSS and UC groups were not matched for time and attention but the therapy duration of UC and RSS were noted after each treatment session (Table 1).

**Table 1:** Baseline characteristics of participants in the repetitive sensory stimulation (RSS) and usual care (UC) groups

	RSS + usual care (*n* = 23)	Usual care (*n* = 17)
Time from stroke to randomisation (days)		
Median (IQR)	4 (3)	6 (8)
Range	2–11	2–14
NIHSS Arm score		
Median (IQR)	1 (3)	2 (3)
Range	1–4	1–4
Dominant hand		
Left	1 (4.3%)	3 (17.6%)
Right	22 (95.7%)	14 (62.4%)
NHSS Arm group		
1–2	16 (70%)	11 (65%)
3–4	7 (30%)	6 (35%)
Age		
Median (IQR)	72.09 (15)	77.15 (21)
Range	37–90	53–92
Gender		
Female	12 (52%)	11 (65%)
Male	11 (48%)	6 (35%)
Pre-stroke Rankin Scale score		
Median (IQR)	1 (2)	2 (2)
Range	0–3	0–3
Total NIHSS score		
Median (IQR)	6 (5)	7 (7)
Range	2–17	2–27
Amount of physiotherapy received (in min)		
Median (IQR)	580 (1180)	520 ((838)
Range	0–3625	0–1240
Amount of occupational therapy received (in min)		
Median (IQR)	520 (1210)	560 (1260)
Range	70–3470	0–1850
FMA-UE		
Median (IQR)	95 (46)	104 (34)
Range	43–118	36–117
FMA-UE (Section H: Sensation) (maximum score:12)		
Median (IQR)	10 (4)	11 (5.5)
Range	0–12	0–12
ARAT		
Median (IQR)	26 (47)	39 (54)
Range	0–57	0–57
NHPT Time (s)		
Median (IQR)	300 (151)	300 (259)
Range	29–300	27–300

*NIHSS* National Institute of Health Stroke Scale, *IQR* interquartile range, *FMA-UE* Fugl-Meyer Assessment Upper Extremity, *ARAT* Action Research Arm Test, *NHPT* Nine-hole peg test, if participants could not undertake the test, they were scored as taking 300 s (maximum time allocated before terminating the test)

Acceptability was evaluated by sending 21 participants/carers in the RSS group at Chester a postal questionnaire after the completion of the study. The questionnaire was developed specifically for this study and comprised 10 open, free-text questions (see Additional file 1). These were completed by the participant and/or their carer and asked about: their perception of the RSS glove, ease of use, positive and negative aspects of using it, whether they felt it helped, if so how, what they did when wearing it, would they recommend it to others and would they use it again in future plus provide any other comments about their experience.

#### Measurements

Demographic data comprising type of stroke (ischaemic or haemorrhagic), pre-stroke and immediate post-stroke function (mRS), stroke severity (NIHSS) were collected on all participants prior to commencement of the study. Outcome tools that were anticipated to be the primary indicators of effectiveness in a future trial were used to measured arm function. The anticipated primary outcome tool was the Action Research Arm Test (ARAT). The ARAT is a 19-item observational tool. Items are categorised into four subscales (grasp, grip, pinch and gross movement) with increasing difficulty. A participant’s performance is rated on a 4-point scale, ranging from 0 (no movement) to 3 (movement performed normally). It is well-validated in stroke rehabilitation and is recommended as the key functional outcome tool of arm activities after stroke by an international, multi-disciplinary expert group [26]. Total scores either indicate no upper limb capacity (0–10), poor capacity (11–21), limited capacity (22–42), notable capacity (43–54) or full capacity (55–57) [30, 31]. The ARAT was assessed by an independent blinded assessor by viewing the video recording of the participants performing completing this test both at 2 weeks and at the end of 3 months from the randomisation [32].

Secondary anticipated outcomes included the Fugl-Meyer Assessment of upper extremity outcome tool (FMA-UE) which is a well-recognised and recommended observational measure of upper limb impairment [26]. This test comprises four sections for the upper limb and each of the 33 items is a scored on a 3-point Likert scale (0 = cannot perform to 3 = performs fully). A maximum score of 66 indicates full upper limb capacity, a score between 48 to 53 indicates notable capacity, 32 to 47 limited capacity, 23 to 31 poor capacity and 0 to 22 no capacity. The time taken to complete the nine-hole peg test (NHPT) was also used to indicate dexterity [32, 33]. These outcomes were re-assessed 2 weeks after starting the intervention and at 3 months’ follow-up by a blinded assessor who viewed video recordings of participants completing the items on each outcome tool.

### Analysis

Feasibility was evaluated by examining:

- Ease of recruitment expressed as a proportion of enrolled participants/proportion of the screened participants from inpatients on the stroke units
- Adherence of using the RSS glove (expressed as a percentage of the maximum time of 630 min if the glove was worn for 45 min, every day for 2 weeks). This was collected both manually by asking patients or their family member to complete a daily treatment diary during the treatment period which was subsequently compared with the data downloaded from the RSS generator, so was not reliant on participant recollection. Reasons for non-adherence were collected, where possible.
- Safety of the intervention over 3 months (including the 2-week intervention period). Several potential adverse events were specifically identified and were:
  - ◦ any damage to the skin integrity of the hand (including ulcers, necrosis) within 30 days of enrolment
  - ◦ epileptic seizures
  - ◦ the presence of a painful shoulder on the affected upper limb
  - ◦ contracture of the affected hand, and
  - ◦ any other adverse events reported by the investigator

Acceptability was judged from RSS participant’s responses to the postal questionnaire sent after the study had finished.

Changes in the anticipated primary and secondary outcome measures for a future trial were examined using descriptive statistics; in this application non-parametric methods were used due to the data being not normally distributed. Logistic regression was also used to assess the association between intervention and outcome, both directly and adjusted for baseline ARAT scores. It should be noted that as this is pilot study the study is not powered to detect differences in outcome measures and as a consequence no formal hypothesis testing is undertaken. The changes in scores in the RSS and UC groups were compared to published values of minimal clinical important differences (MCID) in acute and chronic stroke [30, 31, 34–36]. A pre-specified set of criteria of ‘successful outcome’ for the primary outcome measure (ARAT) was developed based on the improvement in the ARAT score used by Shaw et al. in BoTULS trial [37]. A successful outcome was defined as:

- ≥ 3 points’ improvement if baseline ARAT score of 0–3
- ≥ 6 points’ improvement if baseline ARAT score of 4–51
- An ARAT score of 57 or above if baseline ARAT score was > 51

Using these pre-specified criteria of good outcome, a calculation using a 80% power was used to indicate the sample size needed to detect an increase in the proportion of good outcomes from 45% to 57.5% with treatment (α = 0.05, two-tailed) [38]. All data were analysed using IBM SPSS software version 24.

## Results

### Feasibility

**Fig. 2:**
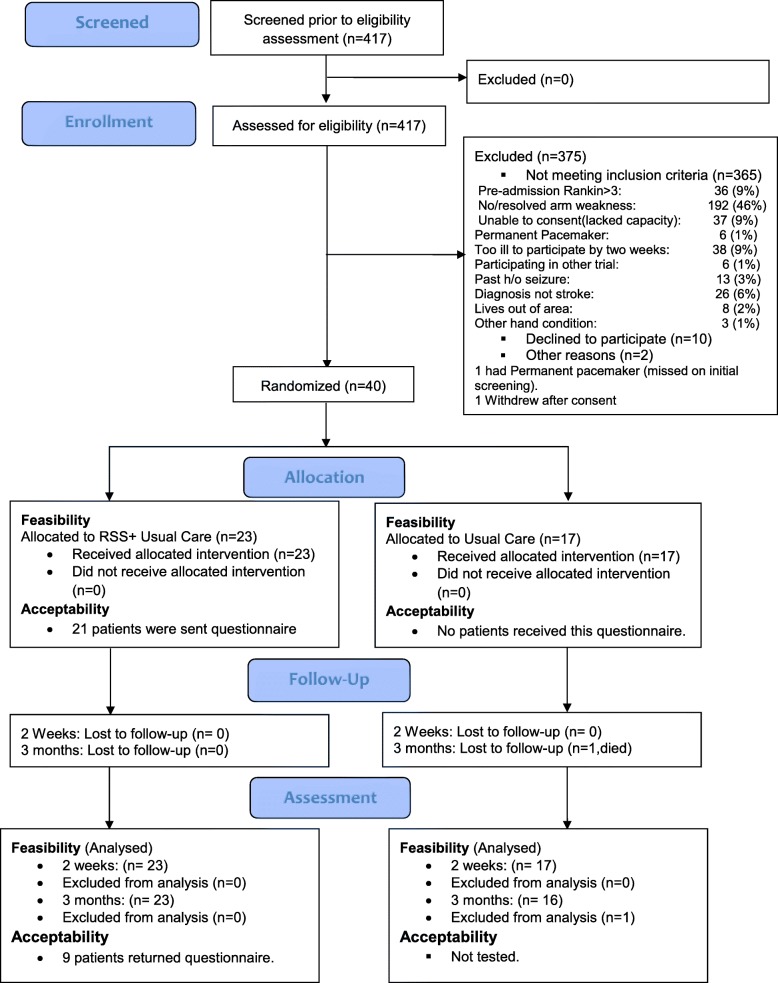
Consolidated Standards of Reporting Trials (CONSORT) diagram showing the flow of participants through each stage of the trial

From 9 January 2017 to 10 November 2017 (10 months), 417 people admitted after a stroke were screened and 52 of them were eligible to participate; of which 40 (23 women, four left-handed) were included in the trial, giving a recruitment rate of 77% of those eligible to participate. The reasons for exclusions non-recruitment and participant flow through the study are illustrated in Fig. 2.

After providing consent, participants were randomised to usual care (UC, *n* = 17) or RSS groups (RSS, *n* = 23). All participants had suffered an ischaemic stroke except one who had a haemorrhagic stroke and was randomised to the RSS group. Twenty-eight participants had known hypertension (RSS = 16, UC = 12), 12 had a history of atrial fibrillation (AF) (RSS = 5, UC = 2) and five had survived a previous stroke (RSS = 3, UC = 2). Seven participants had a stroke affecting their dominant side in the RSS group, with eight having a stroke on their dominant side in the UC group. Baseline characteristics of participants in the RSS and UC groups are shown in Table 1.

Both groups received over 18 h of therapy during the intervention but, as groups were not matched for time and attention, the RSS group received somewhat more (combined occupational and physio therapy; RSS median, range 1305, 70–7095 min; UC 1085; 0–3380 min). However, the amount of upper-limb-specific physiotherapy time was not different between the groups (combined upper limb physiotherapy; RSS median, range 210, 135–335 min; UC 215; 0–445 min).

Adherence to the RSS intervention appeared good. Eleven participants (48%) completed 45 min in every session and so received the maximum dose of RSS (630 min, 100%) and a further eight (35%) received over 75% of the maximum dose (over 495 min). Only two participants (4%) completed less than 50% of the sessions as they had had carotid surgery (endarterectomy) during the study period. Other reasons for non-completion of all the sessions was machine dysfunction (*n* = 2, 9%), and patient choice (*n* = 6, 26%).

Few adverse events (Table 2) were recorded and were generally mild. Shoulder pain was reported by eight (35%) people in the RSS group and five (29%) in the UC group. One person in the RSS group reported pain in the web space of their thumb which had been there since having their stroke and was not worsened by the intervention. No participants had any seizures, but one participant in the UC died during the study period.

**Table 2:** Adverse events

	RSS + Standard treatment	Standard treatment
Shoulder pain		
No	15 (65%)	12 (71%)
Yes	8 (35%)	5 (29%)
Hand pain		
No	22 (96%)	17 (100.0%)
Yes	1 (4%)	

*RSS* repetitive sensory stimulation

#### Acceptability to participants

Nine participants and/or their carers from the RSS group completed and returned questionnaires (return rate 43% of 21 participants). Two reported that they found the RSS glove easy to use, seven found it ‘fiddly’ initially but six of these seven reported that this got easier with practice. Three participants felt that the glove had not worked, but three participants felt that they had more movement in their hand after the intervention. Five participants reported no negative effects after using the glove, two felt that it was slightly painful and the remaining two respondents reported that it was quite tight with one noting that their skin appeared dry after using it. Five people would recommend the RSS glove to other people who have had a stroke whilst one would not. Two would recommend it if it was shown to be beneficial, whilst one participant stated that it was worth trying.

### Outcome

Changes from baseline to 2 weeks and 3 months on the outcome measures used are presented in Table 3 (please see Additional file 3 for anonymised dataset). A change in the ARAT scores indicating a successful outcome from baseline was seen for 16 (70%) people in the RSS group and eight (47%) in the UC group at 2 weeks. At 3 months, this increased in both groups (RSS 17 people, 74%; UC 11 people, 69%).

**Table 3:** Outcomes

Outcome tool	2 weeks		3 months	
	RSS (*n* = 23)	UC (*n* = 17)	RSS (*n* = 23)	UC^a^ (*n* = 16)
Change in ARAT				
Median (IQR)	8 (19)	3 (16)	16 (30)	7 (23)
Range	0–38	− 9–30	0–51	0–55
Change in FMA-UE				
Median (IQR)	12 (15)	6 (12)	16 (14)	11.5 (13)
Range	− 1–33	− 11–22	− 6–45	− 6–31
Change in NHPT^b^				
Median (IQR)	− 6 (− 162)	0 (− 82)	− 55 (− 163)	− 9 (− 206)
Range	− 282–17	− 252–26.1	− 269–0	− 265–11.8

^a^indicates *n* = 16 as 1 participant in the UC group died before 3 months. Positive changes indicate improvement except for NHPT. *ARAT* Action Research Arm Test, *RSS* repetitive sensory stimulation, *UC* usual care, *FMA-UE* Fugl-Meyer Assessment Upper Extremity, *IQR* interquartile range, *NHPT* nine-hole peg test, ^b^if participants could not undertake the test, they were scored as taking 300 s

To further quantify the improvement in outcome after using the intervention, logistic regression was used to calculate an odds ratio that showed that after using the glove the patient was over three times more likely to reach a good outcome at 2 weeks (odds ratio (OR) = 3.27, 95% confidence interval (0.88, 12.13)) and 1.5 times at 3 months (OR = 1.55, 95% confidence interval (0.44, 5.53)). As those in the intervention group had a lower baseline ARAT score, this variable was then added to the model to estimate an adjusted odds ratio. After adjusting for baseline ARAT score those patients who used the glove were still over three times more likely to achieve a good outcome at 2 weeks (adjusted OR = 3.10, 95% confidence interval (0.79, 11.39)) and 1.3 times at 3 months (adjusted OR = 1.36, 95% confidence interval 0.37, 5.02)). These differences are not statistically significant, but the study was not powered to detect a statistically significant difference. However, it provides sufficient evidence of efficacy to go forward to a definitive trial.

**Fig. 3:**
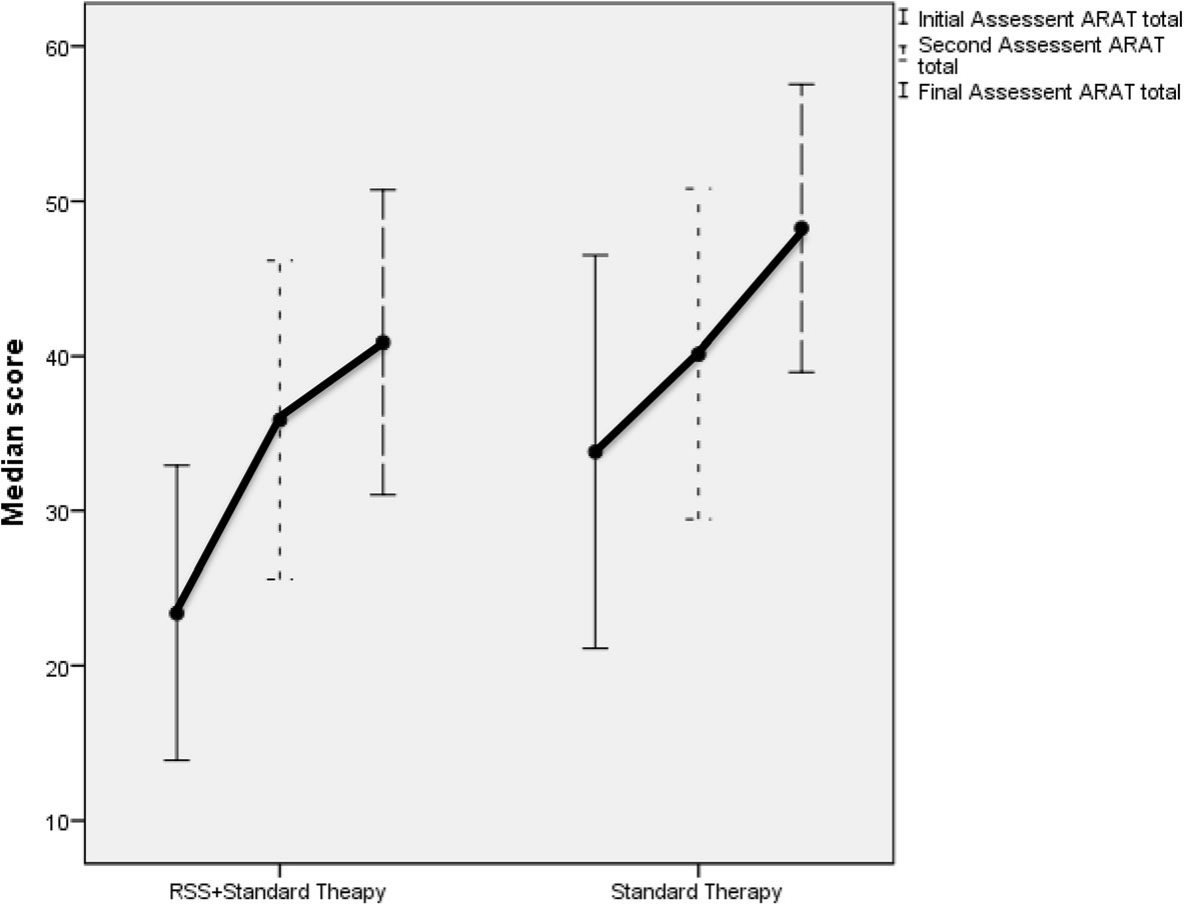
Error plot of change of median Action Research Arm Test score with time in both groups

**Fig. 4:**
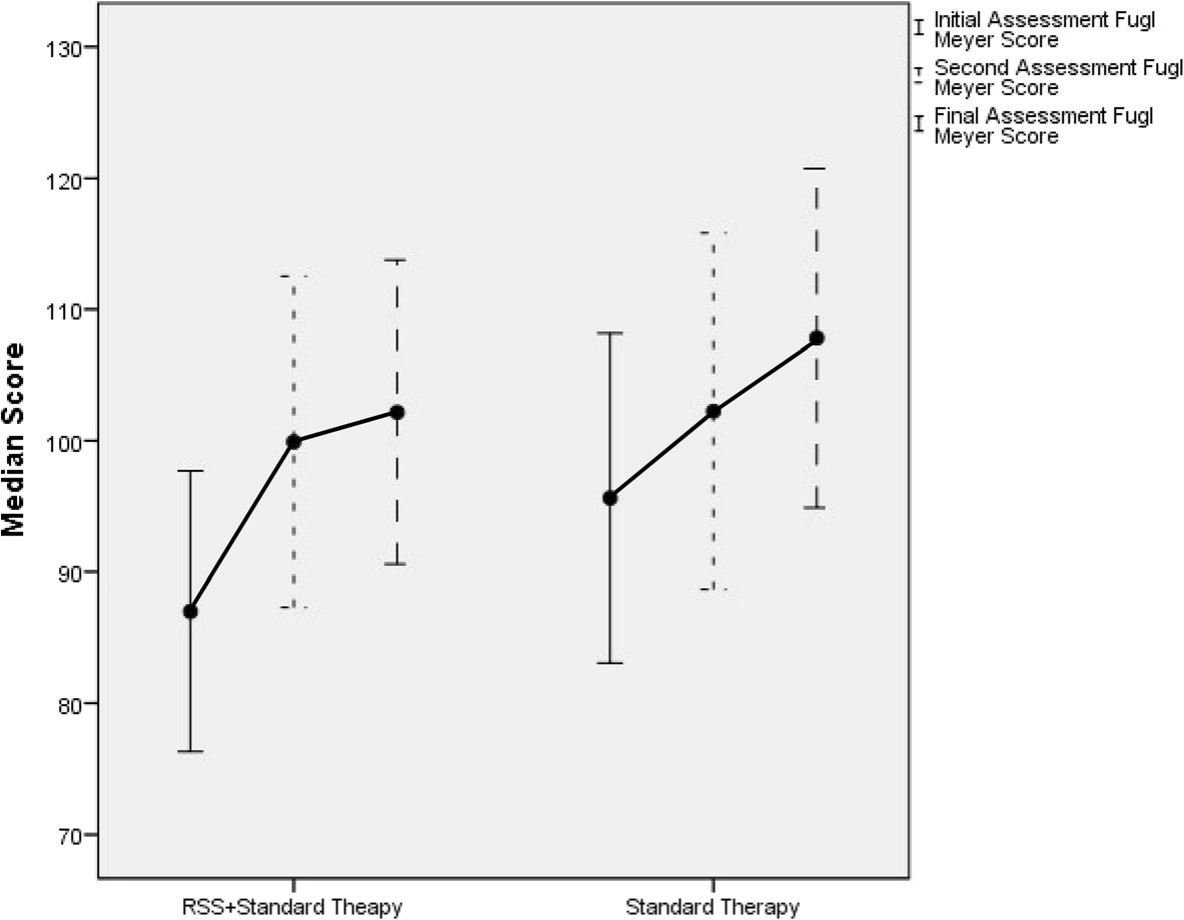
Error plot of change of Median Fugl-Meyer score with time in both groups

The change in median ARAT and FME-UE scores in both groups are illustrated in Figs. 3 and 4 (for waterfall plots on individual changes in ARAT score please see the Additional file 2: Figure S1A and S1B) In the RSS group, 10 participants (from 23, 44%) had a change exceeding the minimal clinical important difference (MCID, 12 points) in acute stroke [31] compared to four people (from 17, 24%) in the UC group exceeded the MCID at 2 weeks. At 3 months, the number of people in the RSS group who exceeded the MCID of 5.7 points on the ARAT for chronic stroke [30] increased to 16 (70%) and to nine in the UC group (56%).

The FMA-UE scores showed that 13 people in the RSS group exceeded the MCID of 9 points at 2 weeks compared to four in the UC group [39, 40]. At 3 months, 15 people in the RSS group and nine in the UC group had improved by over 9 points. On the NHPT, 10 participants exceeded the minimal detectable change of 33 s in the RSS group, compared to five in the UC group; these values were unchanged at 3 months [40].

Based on these results, the sample size needed for a definitive trial of effectiveness of RSS in the acute period after stroke will be 550 participants, including a 10% attrition rate. A trial with 247 patients per group would have 80% power to detect an increase in the proportion of positive clinical outcomes from 45% to 57.5% using the intervention (α = 0.05, two-tailed) [38]. If we upped the power to 90% then we would require 331 per group and again allowing for 10% attrition a total sample size of 736 would be required to detect an increase of 12.5% in the proportion of positive clinical outcomes.

The full trial will use a centralised, web-based, computerised, randomisation system usually used by our clinical trial unit based at the University of Central Lancashire.

## Discussion

This is the first study to examine an RSS intervention in the acute, very early period after stroke and provides important data regarding the feasibility of a trial of RSS and the safety and acceptability of the RSS intervention during this time. The rehabilitation undertaken in the first few days and weeks after stroke is likely to be immensely influential on long-term outcomes [41, 42]. The finding of this study indicate that the RSS intervention delivered in the acute and early subacute phases after stroke [21] appears safe and acceptable and may benefit upper limb function when used to augment usual care.

### Feasibility and acceptability

The first aim of the study was to examine the feasibility of an RSS intervention during the early acute period after stroke. The majority of inpatients after stroke did not conform to the inclusion criteria (*n* = 365) or declined to participate (*n* = 10) resulting in a recruitment rate of 11%. The largest number of potential participants were excluded as they had no or very mild arm involvement after stroke and/or had significant functional restrictions prior to having their stroke. Other trials using forms of RSS have reported some challenges in recruiting suitable participants [21]. This may have been exacerbated in the current study as participants were approached in the first days after stroke and so may have been more likely to decline to participate whilst others were unable to clearly give informed consent due to cognitive or communication problems (*n* = 37). These findings indicate that for a future trial of RSS, a multi-centred design will be required to ensure that the study is adequately powered and that different formats of presenting information and gaining consent for those with communication difficulties and/or cognitive problems should be considered to broaden inclusion.

Once recruited to the study, adherence to the use of the RSS glove was good with 19 from 23 participants completing over 75% of the entire treatment dose. There were few adverse events and those that were reported were relatively minor (dry skin, shoulder discomfort). There were no drop outs from the RSS group, suggesting that the intervention was well tolerated. From those that returned the questionnaire, participants found the glove relatively easy to use after practice and familiarisation and most reported benefit. These findings agree with other reports of RSS in subacute stroke [21] and indicate that RSS may be an attractive treatment for people early after stroke but are limited as a standardised tool to quantify acceptability was not used, and it is not known why less than half of those asked returned their questionnaires and hence this part of the result should be interpreted with some caution.

### Changes after the intervention

The ARAT, FMA-UE and 9HPT all indicated somewhat greater improvement in the RSS group when compared to the UC group, with benefits exceeding the MCID for the majority of RSS participants on the ARAT and FMA-UE. These outcomes were chosen as they are recommended by a recent roundtable for stroke rehabilitation experts and have demonstrated excellent validity, reliability and responsiveness to rehabilitation interventions for the upper limb after stroke [26]. Improvements on all outcomes were most marked immediately after the intervention period and the rate of improvement appeared to attenuate after the intervention had ceased. This might indicate that an intervention period longer than 2 weeks used in the current study might elicit even greater improvements. Few have used an intervention period of more than 2 weeks when evaluating RSS. Peurala et al. (2002) applied RSS twice a day for 3 weeks in 59 people with chronic stroke; Conforto et al. (2010) used it three times a week for 1 month in 22 people with subacute and chronic stroke whilst participants in Smith et al.’s (2009) study received 9 min of sensory stimulation four times a week for 6 weeks [15, 18, 43]. Whilst all reported some improvements in upper limb function, none used similar outcome measures either to each other or to the current study, making direct conclusions about the effects of dose impossible [44]. This indicates that future studies should consider the effect of dose on response to inform the clinical use of RSS.

### Limitations

A key limitation of this study was that the RSS and UC groups were not matched for time and attention and so the differences between groups may be simply attributable to a greater dose of therapy or the effect of more time spent with a health professional in the RSS group, and unrelated to the intervention content. The RSS group received over 165 min more treatment (RSS and usual care) than the UC group during the intervention period and the treatment received after the intervention had finished was not standardised nor monitored in either group. Others have shown that more intensive treatments can elicit greater improvements in upper limb function [43] and it has been suggested that this may be independent of the content of the intervention to some degree [42, 45]. In their randomised controlled trial of RSS in the acute/sub-acute period after stroke, Kattenstroth et al. (2018) included a time-matched control intervention (sham) and found that there were very few significant differences between groups on individual outcome measures, including the NHPT used in the current study [21]. This highlights that inclusion of an appropriate sham treatment is vital in a future trial of RSS to ensure that treatment times and expectations of benefit are as closely matched as possible so that the presence and magnitude of any effect of RSS can be clearly identified.

Another limitation of the current study was that the groups were not fully equivalent at baseline. The RSS group was randomised to start their treatment 2 days earlier than the UC group, and the RSS group had slightly better function prior to their stroke (median pre-stroke mRS scores, interquartile range (IQR) RSS = 1, 2; UC = 2, 2) and marginally better stroke status (median NIHSS scores, IQR RSS:6, 5; UC 7, 7). However, despite stratification on NIHSS arm scores the RSS group demonstrated poorer arm function at baseline (ARAT FMA-UE), suggesting that the NIHSS arm score may not be sensitive or suitable to stratify groups in a definitive trial. Other tools which could be used to stratify groups to ensure equality in a future trial include the SAFE score and/or PREP2 algorithm [46, 47]. These tools have shown an ability to predict the recovery of arm function in 75% of people after stroke but are complicated by their need to use transcranial magnetic stimulation, which may not be available in some clinical settings [48].

Despite potential practical limitations, exploration of sensorimotor cortical function does have an important role in providing an understanding of the mechanisms by which RSS may elicit changes in upper limb function after stroke. Some have reported specific reductions in GABA-ergically mediated intra-cortical inhibition in the motor cortex which can be present even after a single 2-h RSS session in people with chronic stroke (*n* = 9) [48] whilst others have found that a longer 4-week duration of thrice weekly RSS in people with chronic stroke did not significantly alter corticomotor excitability from baseline [18]. These findings are supported by others [19] and indicate that the primary mechanism of RSS is likely to be potentiation via glutamatergic connections between the primary sensory and motor cortices, rather than alterations in intra-cortical excitability [14]. However, further research is needed to inform an ‘optimum’ dose of RSS to benefit motor function after stroke as inconsistencies in the data mean that there is little evidence on which to base current treatment parameters.

## Conclusions

The results from this single-blinded, randomised controlled feasibility study show that RSS is acceptable to use in the early acute period after stroke and that recruitment to a trial to determine its effectiveness is feasible but is likely to require a multi-centre design. This is the first study of RSS in the acute period after stroke and showed that an RSS intervention was well tolerated and that participants were largely adherent to the daily RSS programme over 2 weeks. The differences between groups at baseline suggest that a definitive trial of the effectiveness of RSS for people in the early period after stroke should consider using a more sensitive measure of arm function and/or a prognostic indicator to stratify groups to ensure equality. The RSS intervention appeared to elicit a tendency towards larger improvements during the intervention period than usual care alone, but groups were not matched for time and attention and the trial was too small to identify any significant statistical difference. Therefore, a future trial should include a credible control intervention, such as a sham glove. The differences between the measures of upper limb function between the UC and RSS groups were most marked during the intervention and were attenuated at 3 months. Whilst many studies have used a shorter intervention period than the current work, these findings suggest that further research is necessary to determine if a longer or more intensive programme of RSS could elicit larger changes in upper limb function than those seen here and to elucidate the mechanism by which RSS may improve sensorimotor function.

## Additional files

Additional file 1: PULSE feedback questionnaire for participants and their carers. (DOCX 27 kb)

Additional file 2: **Figure S1.** A: Waterfall plot: changes in the Action Research Arm Test (ARAT) score in the repetitive sensory stimulation (RSS) group at 3 months compared to baseline. B: Waterfall plot: changes in the ARAT score in the standard therapy group at 3 months compared to baseline. (ZIP 340 kb)

Additional file 3: Anonymised data used during the analysis. (XLSX 12 kb)

## Data Availability

All data analysed during this study are included in this published article (as supplementary information files).

## Abbreviations

ADLs: Activities of Daily Living
ARAT: Action Research Arm Test
FMA-UE: Fugl-Meyer Assessment of upper extremity
MCID: Minimal clinical important differences
mRS: Modified Rankin Scale
NHPT: Nine-hole peg test
NIHSS: National Institute of Health Stroke Scale
OR: Odds ratio
RSS: Repetitive sensory stimulation
RTT: Repetitive task training
UC: Usual care
