# Supplementary material for: PULSE-I - Is rePetitive Upper Limb SEnsory stimulation early after stroke feasible and acceptable? A stratified single-blinded randomised controlled feasibility study
Source: Trials. 2019 Jul 1;20:388. doi: 10.1186/s13063-019-3428-y (PMC6604268; doi:10.1186/s13063-019-3428-y)
Supplement: Supplementary file 1 — PULSE feedback questionnaire for participants and their carers. (DOCX 27 kb) [file 13063_2019_3428_MOESM1_ESM.docx]

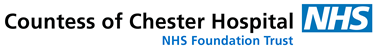


| P.U.L.S.E. Questionnaire for recruited people and their carers  Your observations and suggestions are always very important to us; could you put your own personal comments in the separate boxes below, describing your impressions during the use of the glove. | | |
| --- | --- | --- |
| **Question** | **User comments** | **Carer comments** |
| Can you tell us the main reasons why you wanted to join the PULSE research? |  |  |
| How easy was the Tipstim glove to get on and off, and generally how was it during the daily period of stimulation? |  |  |
| Did you find the Tipstim therapy helped? Tell us a bit about how soon you noticed changes, and exactly what differences you did notice? |  |  |
| Did you manage to carry on with normal activities – reading, watching TV etc., whilst wearing the glove? |  |  |
| What were the positive aspects of using the glove-  Why did you like it? |  |  |
| What were the negative aspects of wearing the glove-  Why didn’t you like wearing it? |  |  |
| What other things about yourself/your partner changed whilst using the glove? For example- was there any change in mood or how they were feeling?  Were there any differences in physical aspects- movement, stiffness, mobility?  Any difference in the skin sensation – touching or feeling? |  |  |
| Would you recommend this to other people who have experienced a stroke and were left with problems in upper limbs? |  |  |
| If it was offered for long term use, would you take it? |  |  |
| Any other comments? |  |  |
