# Supplementary material for: PULSE-I - Is rePetitive Upper Limb SEnsory stimulation early after stroke feasible and acceptable? A stratified single-blinded randomised controlled feasibility study
Source: Trials. 2019 Jul 1;20:388. doi: 10.1186/s13063-019-3428-y (PMC6604268; doi:10.1186/s13063-019-3428-y)

Supplementary Figure 1B: Waterfall plot: Changes in the ARAT score in the standard therapy group at three months compared to baseline.

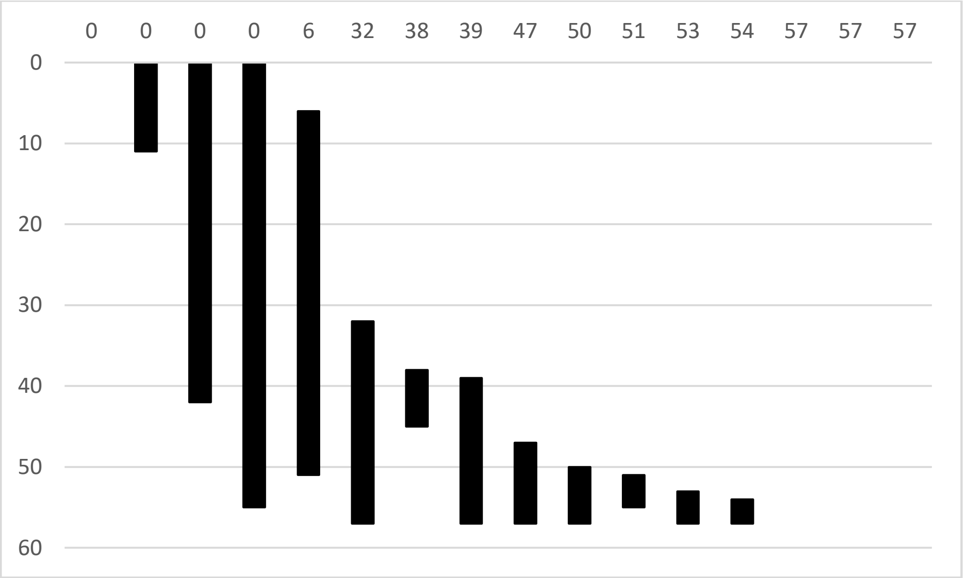

Supplement: Supplementary file 2 — Figure S1. A: Waterfall plot: changes in the Action Research Arm Test (ARAT) score in the repetitive sensory stimulation (RSS) group at 3 months compared to baseline. B: Waterfall plot: changes in the ARAT score in the standard therapy group at 3 months compared to baseline. (ZIP 340 kb) [file 13063_2019_3428_MOESM2_ESM.zip › Supplementary Figure 1BR2.pdf]
